# The Prognostic Significance of the Histological Types in Patients With Nonsmall Cell Lung Cancer ≤2 cm

**DOI:** 10.3389/fsurg.2021.721567

**Published:** 2021-10-25

**Authors:** Bo Hao, Tao Fan, Juan Xiong, Lin Zhang, Zilong Lu, Bohao Liu, Heng Meng, Ruyuan He, Ning Li, Qing Geng

**Affiliations:** Department of Thoracic Surgery, Renmin Hospital of Wuhan University, Wuhan, China

**Keywords:** lung squamous cell carcinoma, lung adenocarcinoma, overall survival, lung cancer-specific mortality, histology

## Abstract

**Background:** Few studies attempt to investigate the impact of histology on the outcome of nonsmall-cell lung cancer (NSCLC) patients. In this study, we aim to determine whether the type of histology influenced the outcome of stage IA NSCLC patients with tumor size (TS) ≤20 mm.

**Methods:** The data of the population in our study was collected from the Surveillance, Epidemiology, and End Results (SEER) program, which is supported by the National Cancer Institute of the United States. The primary outcome was overall survival (OS). Cox-regression proportional hazards models were performed to identify prognostic factors for OS. The secondary outcome was lung cancer-specific mortality (LCSM). A competing risk model was used to identify risk factors associated with LCSM.

**Results:** A total of 4,424 eligible patients (T1a-bN0M0) who received sublobar resection [wedge resection (WR) and segmentectomy] were identified and included in the study for further analysis. For patients with TS ≤ 10 mm, multivariate Cox-regression analyses for OS showed that lung squamous cell carcinoma (LUSC) yielded poorer OS compared with lung adenocarcinoma (LUAD), and no difference was observed between LUSC and LUAD for LCSM in competing risk models. For patients with TS > 10 and ≤20 mm, multivariate analyses revealed that LUSC patients experienced poorer OS compared with that of LUAD; the univariate competing risk analysis indicated SCC pathology predicted an increased risk of death from lung cancer, whereas no difference is observed in the multivariate competing analysis. In addition, segmentectomy was associated with longer OS in patients with >10 and ≤20 mm but not in patients with ≤10 mm compared with WR.

**Conclusion:** Our study demonstrated that squamous pathology was associated with the worse OS but not LCSM for patients with ≤20 mm compared with adenocarcinoma. Moreover, segmentectomy when compared to wedge resection appears to be associated with a better prognosis in patients with neoplasm >10 mm, but not in the case of nodule ≤10 mm.

## Introduction

Lung cancer is the leading cause of cancer-associated mortality for patients and the second most commonly diagnosed cancer in 2020 worldwide (1) and thus has been a huge challenge for public health (2). Currently, surgical resection is the only potentially curative treatment for early-stage nonsmall-cell lung cancer (NSCLC). However, the extent of resection remains debated. Subloar resection is reported to achieve a similar survival to lobectomy in early-stage patients (3, 4), and has been gradually accepted for patients with small tumor size or poor pulmonary reserve (5). NSCLC constitutes about 85% of all lung cancer cases, with adenocarcinoma and squamous cell carcinoma accounting for the most proportion.

Currently, the TNM stage is the major factor that needs to be taken into consideration for clinical decisions, and histological subtype is often ignored in IA stage NSCLC patients. Whether histology should play a role in therapeutic decision-making for IA stage NSCLC patients remains controversial. Some studies pointed out that lung squamous cell carcinoma (LUSC) had a better outcome than lung adenocarcinoma (LUAD) (6, 7), whereas other studies demonstrated LUSC was associated with a worse prognosis (8–10). More and more researchers came to realize that prognostic factors and outcomes were quite different between LUAD and LUSC. Therefore, the difference in prognosis between the two types of lung cancer was needed to be well researched.

In the present study, we collected clinical data from the Surveillance, Epidemiology, and End Results (SEER) database to investigate the prognostic effect of histology on the survival of early-stage NSCLC patients. We performed a population-based study using data from the years ranging from 2004 to 2011 to investigate the impact of histology on postoperative survival of early-stage NSCLC patients.

## Methods

### Data Source

The SEER Program is supported by the National Cancer Institute of the United States. It is one of the largest resources of clinical information on cancers. Data from the SEER database has been used in numerous studies to assess the role of prognostic factors in lung cancer (4, 11–14), and this database is recognized as an authoritative source of clinical information, including tumor histology, tumor size, demographics, primary site, pathological stage, survival time, and so on.

### Study Population

The inclusion criteria in our study should meet: (a) pathologically confirmed primary T1N0M0 NSCLC, only squamous cell carcinoma (SCC) and adenocarcinoma with tumor size ≤2 cm; (b) history of surgery, only wedge resection (WR) and segmentectomy were included; (c) no history of chemotherapy treatment before or after surgery; (d) no record of radiation treatment before or after surgery; (e) age ≥50, since LUSC is less likely to occur in patients with an early age; (f) tumor was not located in the main bronchus; (g) active follow-up and follow-up time no less than 3 months.

The study variables in this study included the baseline demographics of the population (gender, age at diagnosis, and race record), the details of tumors (TNM stage, grade, location, size, and histology diagnosis), and surgical procedures (wedge resection and segmentectomy). All patients were divided into two cohorts according to histology (adenocarcinoma and SCC). The histological type of the enrolled cases was identified according to the third edition of the International Classification of Diseases for Oncology (ICD-O-3). The histological types were included as follows: adenocarcinoma (8140–8147, 8244, 8245, 8250–8255, 8260, 8290, 8310, 8320, 8320, 8323, 8330–8332, 8470, 8480–8481, 8550–8551, 8570–8573) and SCC (8052, 8070–8075, 8078, 8083–8084) (15–17). Surgical procedures (SP) were divided into wedge resection (WR) (surgery code: 21) and segmentectomy (surgery code: 22). The grade well/moderate group included grades I and II, and the poor/undifferentiated (UD) included III and IV.

Overall survival and lung cancer-specific mortality (LCSM) are the primary outcomes to be assessed in our study. The length of time from diagnosis to death due to any cause was defined as OS. The length of time from diagnosis to death due to NSCLC was defined as LCSM, and death from causes other than lung cancer was considered a competing risk event. To assess the impact of TS on OS and LCSM, the study populations were further stratified by TS.

### Statistical Analysis

The difference in the distributions of continuous data (age, number of resected regional lymph nodes, and TS) was calculated by Wilcoxon tests and categorical variables (gender, location, laterality, histology, and grade) by the Pearson χ^2^ tests. The Kaplan–Meier method was used to establish the curves of OS and the difference was evaluated by log-rank tests. All comparisons of OS for all prognostic factors were analyzed by Cox proportional hazards models. A Fine-Gray subdistribution hazard model was performed to identify risk factors associated with LCSM. In the model, death from any other cause, but not lung cancer, was recognized as a competing risk event. It is noted that, only when the univariate analysis indicated a significant difference, multivariate analysis was performed then, and generally, the results of multivariate analyses were more reliable than univariate ones.

A two-sided *P* < 0.05 was considered to indicate a statistical difference in all analyses. All of the hazard ratios (HRs) and its 95% confidence intervals (CIs) in Cox models were calculated using SPSS 22.0 (IBM, Armonk, NY) and all of the subdistribution hazard ratios (HRs) and its 95% confidence intervals (CIs) in Fine-Gray model were analyzed by Stata/SE version 26.0 (Stata Corp. LP, College Station, TX). Survival curves were established by R 4.0.1 (R Development Core Team, R Foundation for Statistical Computing, Vienna, Austria).

## Results

### Baseline Characteristics of the Population

After selection, a total of 4,424 patients with NSCLC ≤ 20 mm (only LUAD and LUSC) were included, of whom 3,211 patients were pathologically confirmed LUAD and 1,213 confirmed LUSC. Among the population, 1,746 were male and 2,678 were female. The date of the study population spanned from January 1, 2004, to December 31, 2011. There were 2,140 patients aged between 50 and 75 years and 2,284 patients aged 76 years and older. The median follow-up time for the patients with adenocarcinoma was 69 months and that for squamous cell carcinoma (SCC) was 58 months (data was not shown). The detailed descriptions of variables and the correlation between each variable and histology were presented in Table 1. Compared to patients diagnosed with LUAD, LUSC patients were more likely to occur in the male gender, white origin, and upper lobe. In addition, larger TS and advanced tumor grades were significantly associated with LUSC.

**Table 1:** Baseline characteristics.

	**AD (*n* = 3211)**	**SC (*n* = 1213)**	***P* value**
**Gender**			<0.001
Male	1,233 (38.4%)	599 (49.4%)	
Female	1,978 (61.6%)	614 (59.6%)	
**Age (years)**			<0.001
50–75	1,627 (50.7%)	513 (42.3%)	
≥76	1,584 (42.3%)	700 (57.7%)	
**Race**			<0.001^*^
White	2,827 (88.0%)	1,102 (90.8%)	
Black	233 (7.3%)	85 (7.0%)	
Others	151 (4.7%)	26 (2.1%)	
**Grade**			<0.001^*^
Well/moderate	2,290 (71.3%)	678 (55.9%)	
Poor/UD	555 (17.3%)	465 (38.3%)	
Unknown	366 (11.4%)	70 (5.8%)	
**Resected LNs**			0.039^*^
0	1,506 (46.9%)	603 (49.7%)	
1–3	778 (24.2%)	299 (24.6%)	
≥4	799 (24.9%)	254 (20.9%)	
Unknown	128 (4.0%)	57 (4.7%)	
**Tumor size (mm)**			<0.001^*^
≤10	948 (29.5%)	278 (22.9%)	
11-20	2,263 (70.5%)	1,213 (77.1%)	
**Surgical procedure**			0.138
Wedge resection	2,620 (81.6%)	1,013 (83.5%)	
Segmental resection	2,176 (18.4%)	200 (16.5%)	
**Location**			0.023^*^
Upper	1,901 (59.2%)	776 (64.0%)	
Middle	121 (3.8%)	44 (3.6%)	
Lower	1,147 (35.7%)	383 (31.6%)	
others	42 (1.3%)	10 (0.8%)	
**Laterality**			0.720
Left	1,434 (44.7%)	549 (45.3%)	
Right	1,777 (55.3%)	664 (54.7%)	

* *Indicates that the difference was statistically significant*.

*UD, undifferentiated; LN, lymph node; AD, adenocarcinoma; SCC, squamous cell carcinoma*.

### Survival Analysis

**Figure 1:**
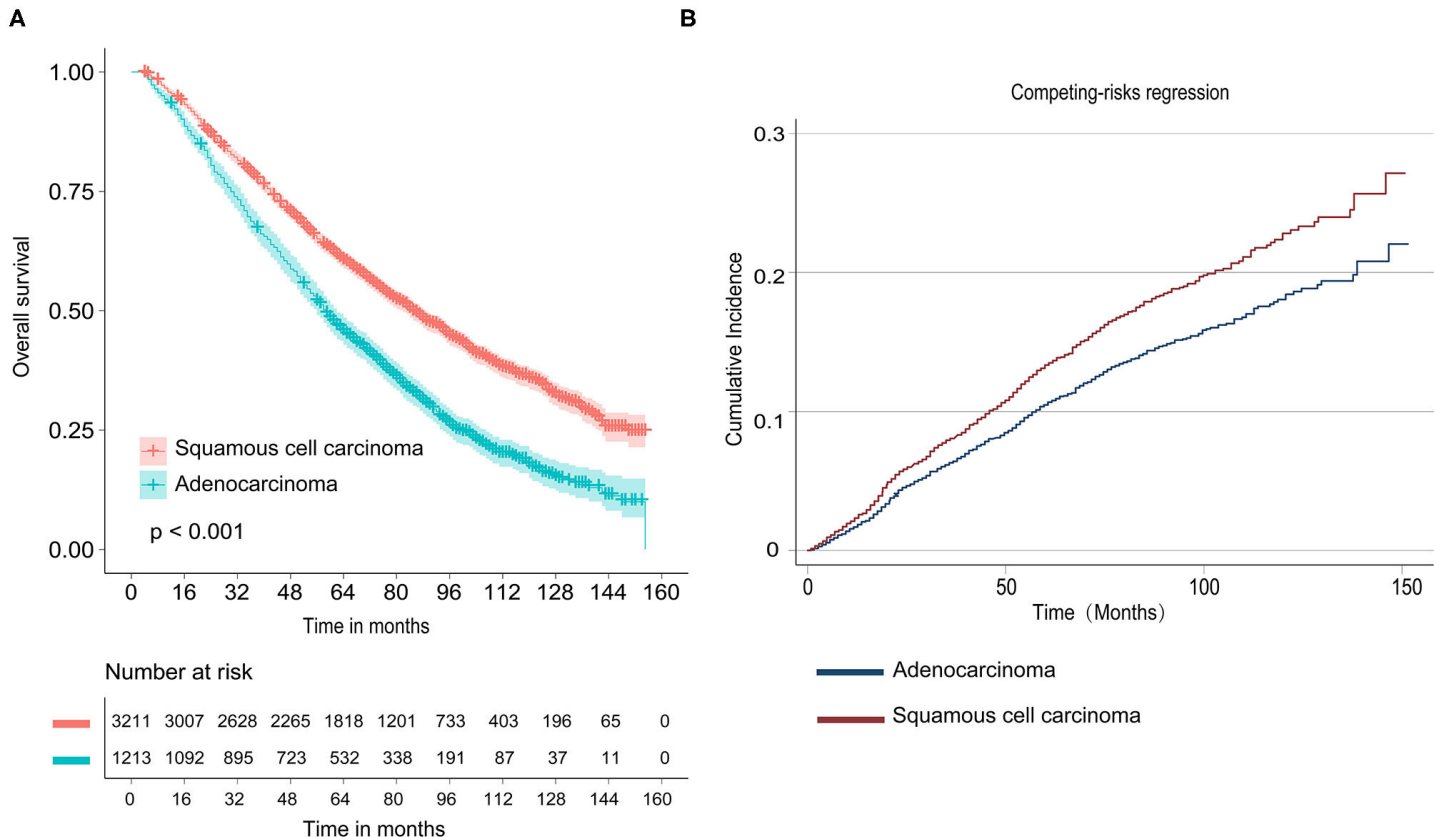
Survival analyses for stage nonsmall-cell lung cancer (NSCLC) patients with tumor size ≤20 mm. **(A)** Kaplan–Meier estimates for overall survival by histological subtype; **(B)** Cumulative incidence for lung cancer-specific mortality by histological subtype.

As shown in Figure 1A, Kaplan–Meier survival curves of OS calculated by log-rank revealed that patients who were diagnosed with adenocarcinoma had better OS than SCC (*P* < 0.001). In Cox-regression proportional hazards models, the results showed that LUSC patients experienced shorter OS [multivariate: HR = 1.367, 95% CI (1.257, 1.285), *P* < 0.001] compared with LUAD (Table 2). We found that patients who received segmentectomy with TS ≤ 20 mm had better OS [multivariate: HR = 0.850, 95% CI (0.763, 0.946), *P* = 0.003] compared with WR. In this study, we also demonstrated that a larger number of resected lymph nodes and smaller TS were strongly associated with longer OS.

**Table 2:** Survival comparisons for NSCLC patients with tumor size ≤20 mm.

	**Overall survival**						**Lung cancer-specific mortality**					
	**HR^a^**	**95% CI^a^**	***P* ^a^**	**HR^b^**	**95% CI^b^**	***P* ^b^**	**SHR^a^**	**95% CI ^a^**	***P* ^a^**	**SHR^b^**	**95% CI ^b^**	***P* ^b^**
**Gender**												
Male	1			1			1			1		
Female	0.682	0.632–0.736	<0.001^*^	0.730	0.676–0.789	<0.001^*^	0.788	0.669–0.929	0.005^*^	0.837	0.708–0.988	0.035^*^
**Age (years)**												
70–75	1			1			1			1		
≥76	1.730	1.601–1.870	<0.001^*^	1.633	1.509–1.767	<0.001^*^	1.251	1.062–1.473	0.007^*^	1.183	1.002–1.396	0.046^*^
**Race**												
White	1			1			1					
Black	0.890	0.763–1.038	0.137	0.950	0.814–1.109	0.707	1.073	0.787–1.461	0.655			
Others	0.677	0.544–0.841	<0.001^*^	0.651	0.523–0.811	<0.001^*^	0.705	0.447–1.111	0.133			
**Location**												
Upper	1						1					
Middle	1.075	0.886–1.306	0.463				1.408	0.972–2.039	0.070			
Lower	0.968	0.892–1.050	0.429				0.862	0.719–1.032	0.107			
Others	1.214	0.868–1.696	0.257				1.084	0.499–2.355	0.838			
**Laterality**												
Left	1						1					
Right	1.004	0.931–1.084	0.911				1.090	0.924–1.286	0.305			
**Grade**												
Well/moderate	1			1			1			1		
Poor/UD	1.344	1.231–1.467	<0.001^*^	1.215	1.109–1.331	<0.001^*^	1.406	1.170–1.690	<0.001^*^	1.348	1.111–1.636	0.002^*^
Unknown	0.984	0.863–1.122	0.806	1.065	0.933–1.216	0.352	0.797	0.580–1.096	0.163	0.855	0.621–1.178	0.339
**Resected LNs**												
0	1			1			1			1		
1–3	0.818	0.745–0.898	<0.001^*^	0.847	0.770–0.932	0.001^*^	0.964	0.792–1.174	0.720	0.939	0.769–1.145	0.534
≥4	0.607	0.549–0.671	<0.001^*^	0.664	0.599–0.736	<0.001^*^	0.752	0.609–0.929	0.008^*^	0.756	0.613–0.933	0.009^*^
Unknown	0.665	0.542–0.816	<0.001^*^	0.673	0.548–0.827	<0.001^*^	0.489	0.285–0.839	0.009^*^	0.473	0.275–0.813	0.007^*^
**Tumor size (mm)**												
≤10	1			1			1			1		
11–20	1.234	1.131–1.347	<0.001^*^	1.176	1.076–1.285	<0.001^*^	1.545	1.260–1.894	<0.001^*^	1.486	1.208–1.826	<0.001^*^
**SP**												
WR	1			1			1					
Segmentectomy	0.758	0.683–0.841	<0.001^*^	0.850	0.763–0.946	0.003^*^	0.930	0.752–1.149	0.503			
**Histology**												
AD	1			1			1					
SCC	1.600	1.476–1.735	<0.001^*^	1.367	1.257–1.285	<0.001^*^	1.274	1.066–1.522	0.007^*^	1.102	0.911–1.332	0.316

a *Univariate analysis*,

b *multivariate analysis*,

* *indicates that the difference was statistically significant*.

*HR, hazard ratio; SHR, subdistribution hazard ratio; UD, undifferentiated; LN, lymph node; AD, adenocarcinoma; SCC, squamous cell carcinoma; WR, wedge resection; LT, lobectomy; SP, surgical procedure*.

As shown in Figure 1B and Table 2, univariate competing risk-regression models for LCSM showed that LUSC patients were more likely to die of lung cancer compared with LUAD [SHR = 1.274, 95% CI (1.066, 1.522), *P* = 0.007]. However, the multivariate analyses indicated the difference was not significant [SHR = 1.102, 95% CI (0.911, 1.132), *P* = 0.316]. Moreover, patients who underwent segmentectomy had a similar LCSM [SHR = 0.930, 95% CI (0.752, 1.149), *P* = 0.503] compared with those who underwent WR.

### Survival Comparisons Stratified by Tumor Size

To further investigate the impact of histology on survival in different TS, the survival analyses were investigated according to subclassification of TS (TS ≤ 10 mm, and TS > 10 mm and ≤20 mm).

**Figure 2:**
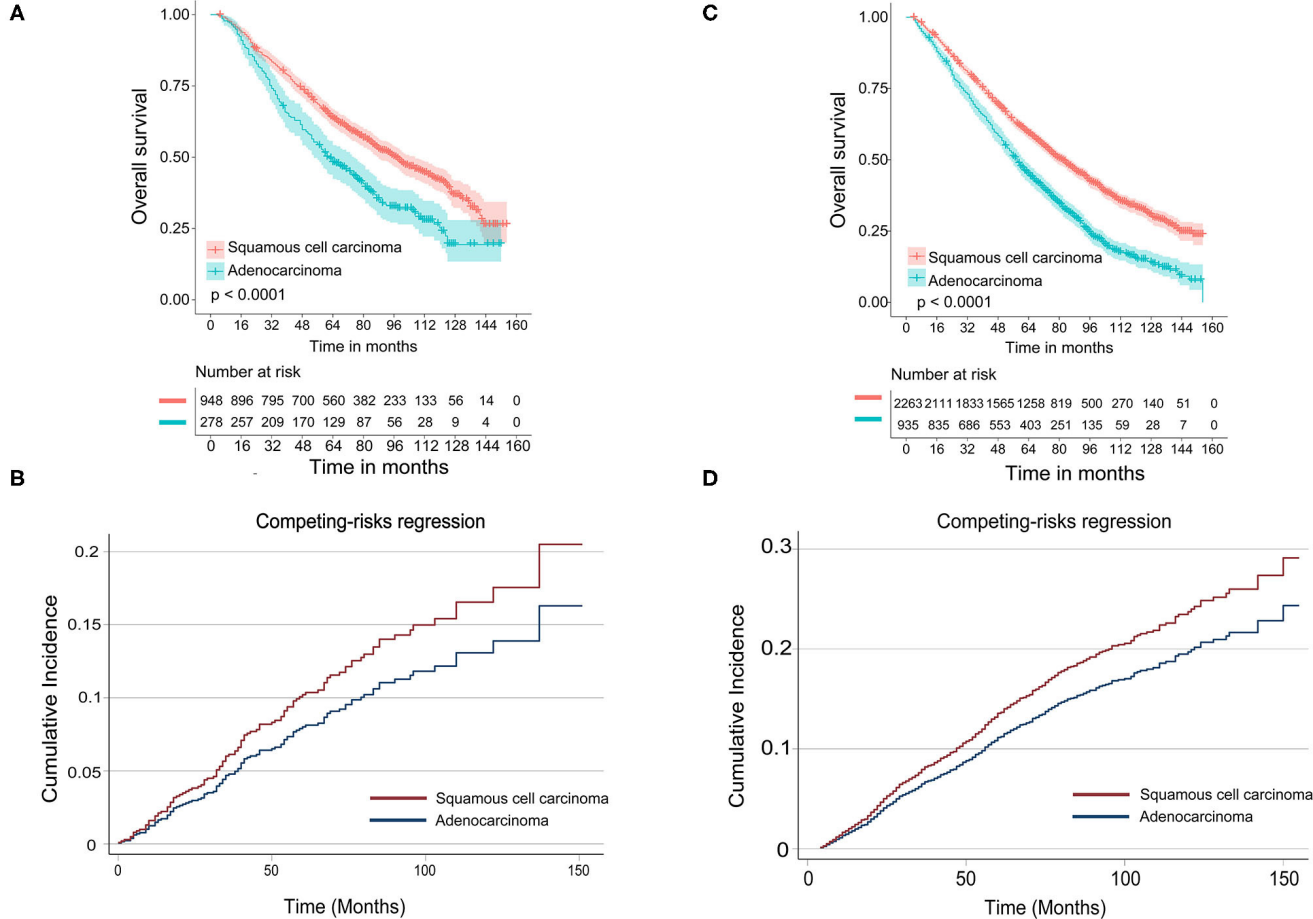
Survival analyses for stage IA NSCLC patients aged 70 and older stratified by tumor size. **(A)** Kaplan–Meier estimates for overall survival by histological subtype of patients with tumor size ≤10 mm; **(B)** cumulative incidence for lung cancer-specific mortality by histological subtype of patients with tumor size ≤10 mm; **(C)** Kaplan–Meier estimates for overall survival by histological subtype of patients with tumor size >10 and ≤20 mm; **(D)** cumulative incidence for lung cancer-specific mortality by histological subtype of patients with tumor size >10 and ≤20 mm.

In the subgroup of TS ≤ 10 mm, there was a significant difference in OS [multivariate: HR = 1.345, 95% CI (1.126, 1.608), *P* < 0.001] between LUAD and LUSC (shown in Table 3), and the survival curves of histology were shown in Figure 2A. In the competing risk model, the difference was not observed in tumor histology and surgical procedures (Table 3 and Figure 2B).

**Table 3:** Survival comparisons for patients with TS ≤10 mm.

	**Overall survival**						**Lung cancer–specific mortality**					
	**HR^a^**	**95% CI ^a^**	***P* ^a^**	**HR^b^**	**95% CI ^b^**	***P* ^b^**	**SHR^a^**	**95% CI ^a^**	***P* ^a^**	**SHR^b^**	**95% CI ^b^**	***P* ^b^**
**Gender**												
Male	1			1			1					
Female	0.668	0.573–0.779	<0.001^*^	0.698	0.597–0.815	<0.001^*^	0.792	0.546–1.149	0.221			
**Age (years)**												
70–75	1			1			1					
≥76	1.652	1.419–1.924	<0.001^*^	1.622	1.392–1.892	<0.001^*^	1.372	0.954–1.972	0.087			
**Race**												
Caucasian	1						1					
African	0.988	0.736–1.326	0.936				1.048	0.529–2.078	0.891			
Others	0.837	0.552–1.270	0.404				0.831	0.308–2.241	0.715			
**Location**												
Upper	1						1					
Middle	0.945	0.635–1.405	0.945				1.146	0.504–2.607	0.744			
Lower	0.973	0.829–1.143	0.743				0.566	0.369–0.868	0.009^*^			
Others	1.179	0.609–2.284	0.625				1.330	0.296–5.981	0.710			
**Laterality**												
Left	1						1					
Right	1.009	0.866–1.176	0.908				0.924	0.642–1.331	0.674			
**Grade**												
Well/moderate	1			1			1					
Poor/UD	1.290	1.068–1.557	0.008^*^	1.156	0.950–1.406	0.147	1.315	0.849–2.036	0.219			
Unknown	0.985	0.788–1.232	0.896	1.100	0.879–1.378	0.405	0.650	0.344–1.228	0.185			
**Resected LNs**												
0	1			1			1					
1–3	0.940	0.780–1.132	0.512	1.003	0.831–1.210	0.978	0.877	0.559–1.374	0.568			
≥4	0.606	0.492–0.747	<0.001^*^	0.661	0.533–0.818	<0.001^*^	0.662	0.405–1.082	0.100			
Unknown	0.627	0.391–1.007	0.053^*^	0.663	0.412–1.066	0.090	0.412	0.103–1.637	0.208			
**SP**												
WR	1			1			1					
Segmentectomy	0.730	0.573–0.930	0.011^*^	0.850	0.663–1.089	0.198	0.819	0.467–1.436	0.487			
**Histology**												
AD	1			1			1					
SC	1.579	1.334–1.869	<0.001^*^	1.345	1.126–1.608	0.001^*^	1.290	0.853–1.951	0.227			

a *Univariate analysis*,

b *Multivariate analysis*,

* *indicates that the difference was statistically significant*.

*HR, hazard ratio; SHR, subdistribution hazard ratio; UD, undifferentiated; LN, lymph node; AD, adenocarcinoma; SCC, squamous cell carcinoma; SR, sublobar resection; LT, lobectomy; SP, surgical procedure*.

As shown in Table 4, in Cox-regression proportional hazard models, SCC predicted worse OS [multivariate: HR = 1.378, 95% CI (1.252, 1.517), *P* < 0.001] in patients with TS >10 mm and ≤20 mm compared with adenocarcinoma. Consistent with the results of Cox-regression analyses, Kaplan–Meier survival curves indicated that SCC was associated with worse OS (*P* < 0.001) (Figure 2C). Besides, segmentectomy achieved better OS [multivariate: HR = 0.850, 95% CI (0.775, 0.957), *P* = 0.007] compared with WR. The difference was also observed in gender, age, the number of resected lymph nodes, and tumor grade. SCC pathology predicted an increased risk of death from lung cancer [SHR = 1.233, 95% CI (1.013, 1.502), *P* = 0.037] in univariate competing risks models (Table 4 and Figure 2D); however, the difference was not significant [SHR = 1.123, 95% CI (0914, 1.382), *P* = 0.265] in multivariate analysis.

**Table 4:** Survival comparisons for patients with tumor size >10 and ≤20 mm.

	**Overall survival**						**Lung cancer-specific mortality**					
	**HR^a^**	**95% CI ^a^**	**P^a^**	**HR^b^**	**95% CI ^b^**	**P^b^**	**SHR^a^**	**95% CI ^a^**	**P^a^**	**SHR^b^**	**95% CI ^b^**	**P^b^**
**Gender**												
Male	1			1			1					
Female	0.696	0.637–0.760	<0.001^*^	0.742	0.679–0.811	<0.001^*^	0.806	0.671–0.969	0.022^*^	0.838	0.696–1.008	0.062
**Age (years)**												
70–75	1			1			1					
≥76	1.732	1.582–1.896	<0.001^*^	1.635	1.491–1.792	<0.001^*^	1.183	0.985–1.421	0.072			
**Race**												
Caucasian	1			1			1					
African	0.859	0.717–1.029	0.099	0.901	0.751–1.080	0.259	1.083	0.766–1.531	0.651			
Others	0.621	0.481–0.802	0.001^*^	0.603	0.466–0.780	<0.001^*^	0.668	0.400–1.114	0.122			
**Location**												
Upper	1						1					
Middle	1.130	0.905–1.412	0.280				1.508	0.998–2.282	0.051			
Lower	0.971	0.884–1.068	0.545				0.966	0.791–1.179	0.732			
Others	1.229	0.833–1.812	0.299				1.010	0.409–2.499	0.982			
**Laterality**												
Left	1						1		1.129			
Right	1.004	0.920–1.097	0.923				1.140	0.947–1.373	0.166			
**Grade**												
Well/moderate	1			1			1			1		
Poor/UD	1.346	1.219–1.487	0.001^*^	1.236	1.115–1.369	<0.001^*^	1.405	1.147–1.720	0.001^*^	1.353	1.094–1.672	0.005^*^
Unknown	1.021	0.867–1.203	0.802	1.050	0.891–1.238	0.562	0.925	0.641–1.336	0.679	0.880	0.608–1.273	0.500
**Resected LNs**												
0	1			1			1			1		
1–3	0.767	0.688–0.854	<0.001^*^	0.798	0.715–0.891	<0.001^*^	0.958	0.769–1.194	0.706	0.943	0.754–1.793	0.609
≥4	0.596	0.532–0.669	<0.001^*^	0.660	0.587–0.743	<0.001^*^	0.756	0.598–0.954	0.019^*^	0.759	0.601–0.959	0.021^*^
Unknown	0.655	0.522–0.822	<0.001^*^	0.674	0.536–0.847	0.001^*^	0.487	0.271–0.877	0.017^*^	0.482	0.267–0.870	0.015^*^
**SP**												
WR	1			1			1					
Segmentectomy	0.745	0.664–0.837	<0.001^*^	0.850	0.755–0.957	0.007^*^	0.912	0.725–1.147	0.434			
**Histology**												
AD	1			1			1					
SC	1.586	1.447–1.739	<0.001^*^	1.378	1.252–1.517	<0.001^*^	1.233	1.013–1.502	0.037^*^	1.125	0.914–1.382	0.265

a *Univariate analysis*,

b *Multivariate analysis*,

* *indicates that the difference was statistically significant*.

*HR, hazard ratio; SHR, subdistribution hazard ratio; UD, undifferentiated; LN, lymph node; AD, adenocarcinoma; SCC, squamous cell carcinoma; WR, wedge resection; SP, surgical procedure*.

## Discussion

In our study, we investigated the relationship between histology types and prognosis of stage I A1–A2 NSCLC patients who underwent sublobar resection. We found that LUSC patients were at a higher risk of reduction of OS compared with LUAD, whereas the difference was not significant for LCSM. After taking TS into full consideration, the results remained stable. These results suggested that the histological subtype might be an independent prognostic factor for OS but not for LCSM in NSCLC patients with TS ≤ 20 mm.

Although numerous studies have involved the relationship between histology subtypes and the prognosis of stage I A NSCLC patients, they all focused on other prognostic factors, and few studies have deeply investigated the impact of histology on the outcome of NSCLC patients. However, the outcome of LUSC and LUAD had mixed results. Some studies suggested that LUSC was associated with a favorable survival (6, 7, 18, 19), whereas other studies pointed out that LUAD yielded better survival than LUSC (8–10), even though there were also studies showing that there were no differences in survival between the two types of histology (20, 21). In our study, patients with squamous histology had a higher risk of shorter OS than those with adenocarcinoma. Nakamura et al. (9) pointed out that LUSC patients were likely to have a history of smoking, and patients with a smoking habit were more susceptible to cancers other than the respiratory system, chronic obstructive pulmonary diseases, pneumonia, ischemic heart diseases, and cerebrovascular diseases, all of which may lead to shorter OS (9). Since some models have inherent weaknesses, it is important to select a suitable model to analyze clinical data. Considering that conventional Kaplan–Meier and Cox models may overestimate the crude incidence of an outcome of interest, competing risk models were used to analyze lung cancer-specific death. The difference in LCSM between LUSC and LUAD was not observed in this study. We speculate that the reason for shorter OS may be that SCC histology is more likely to occur in patients with older age and they die of other causes. Consistent with our results, previous studies suggested that the two types of lung cancer should be analyzed separately to provide more precise outcomes (8, 22).

We demonstrated that segmentectomy achieved better OS for patients with TS > 10 mm and ≤20 mm, but not TS ≤ 10 mm compared with WR, while as for LCSM, segmentectomy yielded a similar outcome compared with WR. These results suggest that segmentectomy is more suitable for NSCLC with TS >10 mm and ≤20 mm than WR. We also found that the number of lymph nodes examined is an important prognostic factor for OS in NSCLC patients who received sublobar resection. A larger number of resected lymph nodes (“4” vs. “0”) was closely associated with longer OS; however, as for LCSM, the difference was significant in patients with TS >10 and ≤20 mm, but not in TS ≤ 10. These results suggested the necessity of examining lymph nodes when an operation was being performed, especially for patients with larger tumor sizes. Recently, a study pointed out that adenocarcinoma and SCC are significantly different in many prognostic factors, such as age, tumor location, smoking status, gender, pathological stage, clinical TNM stage, tumor differentiation grade, and survival (8). Our study also revealed that older age, male gender, advanced tumor grade, and larger tumor size were associated with worse OS.

Certainly, our study has some limitations. In recent years, with the development of targeted therapies and immunotherapies for lung adenocarcinoma, these patients who received targeted therapy and immunotherapy may undergo a longer survival than those who did not. Lacking relative information, we could not further assess the impacts of different targeted therapies and immunotherapy on OS and LCSM. However, IA stage resectable NSCLC patients were less likely to receive such treatments; Therefore, there is little possibility that our study has been substantially affected. Secondly, because of the nature of the retrospective study, some bias was inevitable. Finally, the information of the exact type of resected lymph nodes and the resection margin was not provided, and we will further investigate the impact of these factors on survival in the future. Our results need to be further validated by a large randomized cohort study in the future.

Taken together, our study demonstrated that SCC histology was an independent prognostic factor for the worse OS, but not for LCSM in NSCLC patients with TS ≤ 20 mm who received sublobar resection. Moreover, segmentectomy when compared with WR appears to be associated with a better prognosis in patients with neoplasm >10 mm, but not in the case of nodule ≤10 mm.

## Data Availability Statement

The datasets presented in this study can be found in online repositories. The names of the repository/repositories and accession number(s) can be found in the article/supplementary material.

## Author Contributions

BH, TF, and JX: study design, manuscript writing, and final approval. LZ, ZL, BL, HM, and RH: data collection and analysis. NL and QG: manuscript revision and final approval. All authors contributed to the article and approved the submitted version.

## Funding

This work was supported by the National Natural Science Foundation of China (Grant Nos. 81700093 and 81770095).

## Conflict of Interest

The authors declare that the research was conducted in the absence of any commercial or financial relationships that could be construed as a potential conflict of interest.

## Publisher's Note

All claims expressed in this article are solely those of the authors and do not necessarily represent those of their affiliated organizations, or those of the publisher, the editors and the reviewers. Any product that may be evaluated in this article, or claim that may be made by its manufacturer, is not guaranteed or endorsed by the publisher.
